# Measurement of stopping power ratio of chemo‐ports using energy spectrum extracted from integral depth dose

**DOI:** 10.1002/acm2.70052

**Published:** 2025-02-25

**Authors:** Rosette Gonzalez, Stephen Olis, Sina Mossahebi, Weiguang Yao

**Affiliations:** ^1^ Department of Radiation Oncology University of Maryland School of Medicine Baltimore Maryland USA

**Keywords:** chemo‐port, energy spectrum extraction, stopping power ratio, water equivalent thickness

## Abstract

**Purpose:**

In proton radiotherapy, the stopping power ratio (SPR) of non‐biological materials must be independently measured with proton beams for accurate dose calculation. Small‐size chemo‐ports challenge the measurement. The purpose of this work is to measure the SPR of chemo‐ports by using the energy spectra of the proton pencil beams.

**Methods and materials:**

Chemo‐ports used in this study were irradiated in both lateral and vertical directions by 100‐, 160‐, and 200‐MeV monoenergetic proton pencil beamlets. The integrated depth doses (IDDs) were acquired using a multi‐layer ion chamber (MLIC), with and without the chemo port in front of the MLIC. The energy spectrum (ES) of the IDD was extracted. The water equivalent thickness (WET) of the chemo‐port was determined from the shift in corresponding peaks in the spectra. To reduce the effect of spot size and its Gaussian distribution on the measurement, the measurements were repeated with a lead collimator (5 mm circular opening) in front of the chemo‐ports. Additionally, the WET values were also obtained by a conventional approach that calculated the shift of the peaks in the IDDs rather than in the energy spectra.

**Results:**

The complex internal structure of the chemo‐port was reflected in multiple peaks in the ES. The measured WET values from different energy beamlets agreed within 0.5 mm (4.6%) of each other using the ES method, while agreement up to 1 mm was observed from the traditional approach. When the collimator was used, the agreement was decreased to within 1.1 and 8 mm from the ES method and conventional approach, respectively.

**Conclusion:**

Proton SPRs of chemo ports can be successfully measured using the ES method. Better agreement of the measured WET values from different energy pencil beams was obtained from the ES method than from a conventional approach. The use of a collimator can decrease accuracy.

## 1 INTRODUCTION

Proton radiation therapy is commonly used for treating breast cancer because it can efficiently mitigate breathing effects by using appropriate beam directions,^1^ and minimize the dose to the heart^2^ and lung^3^ compared with conventional photon techniques. Breast cancer patients often go through radiation therapy concurrently with chemotherapy. Chemo‐ports may be located in the proton beam path during the course of treatment and can change the proton range. Since they consist of non‐biological components, their stopping power ratios (SPRs) cannot be reliably converted from the CT number. Therefore, the SPR of chemo‐ports needs to be measured directly from proton beams.

The SPR of a homogeneous material such as a slab of solid water or breast implants can be determined accurately using the measured water‐equivalent thickness (WET) and the physical thickness. The WET is typically measured using a multi‐layer ion chamber (MLIC) from the shift in the range of a pencil beamlet with and without passing through the object. This method is naturally employed to determine the WET of a chemo‐port. However, chemo‐ports consist of multiple materials arranged in a complex internal structure. Additionally, the size of a chemo‐port is small (about 1–2 cm), similar to the full width at half maximum of the spot of a pencil beam. Therefore, the IDD curve acquired with a chemo‐port will have a different and complicated shape, making it difficult to determine the range shift of the proton beam. One way to improve the measurement is to reduce the spot size of a pencil beamlet by adding a collimator with a very small opening in front of the chemo‐port. In this case, because of the divergence of the pencil beamlet, a part of the protons will interact with the collimator wall resulting in a change of the shape of IDD.

The purpose of this work is to improve the measurement of the WET of chemo‐ports. Instead of observing the shift of the range with and without the chemo‐port, we extracted the proton energy spectrum (ES) from measured IDD,^4^ and then observed the shift of the corresponding peak in the ES to determine the WET of the chemo‐port.

## 2 METHOD AND MATERIALS

### 2.1 Characteristics of the chemo‐ports

**FIGURE 1:**
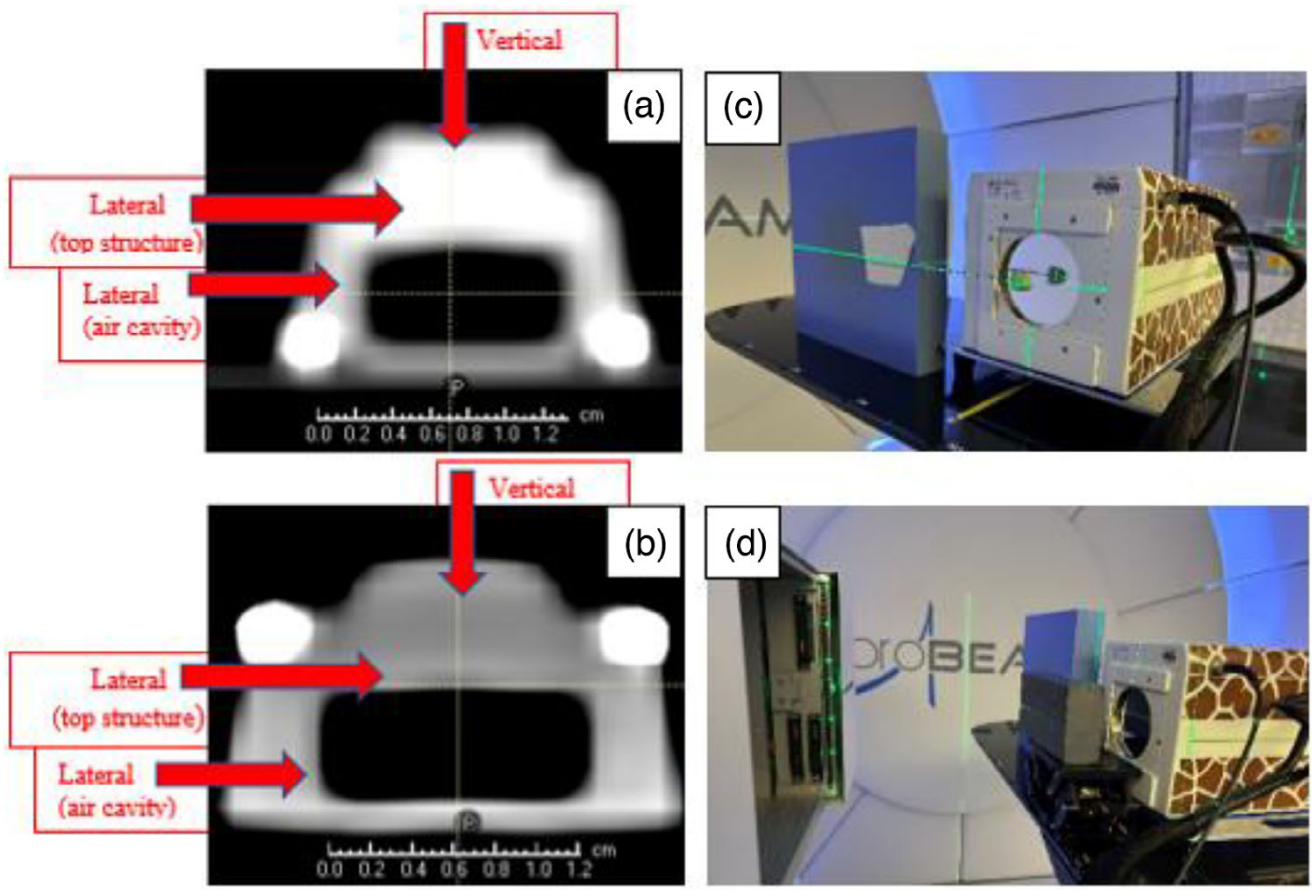
CT image of two types of chemo‐ports and the measurement setup for acquiring the integrated depth doses (IDDs) with and without a collimator. A stick attached to the stem of the chemo‐port is used to adjust the orientation of the chemo‐port easily. The red arrows indicate the beamlet directions, vertical and lateral to the chemo‐port. (a) slim port, (b) isp port, (c) setup without a collimator, and (d) setup with a collimator.

Two types of chemo‐ports, PowerPort ClearVUE isp Implantable Port and PowerPort ClearVUE Slim Implantable Port (BARD Access Systems, Boston, USA), were used in this study.^5^ Both chemo‐ports are metal‐free in construction, and have a lightweight design body. The slim or small implantable port is a low profile created with plastic and uses silicone‐filled and non‐filled suture holes for the suture mechanism.^6^ The isp or big implantable port is an intermediate‐sized one created with plastic and uses a silicone over‐mold suture mechanism.^7^ The size of slim port (see Figure 1a) is 28 mm of base diameter, 10.4 mm of height and the internal volume is 0.4 mL and that of isp port (Figure 1b) is 29 mm of base diameter, 11.9 mm of height and the internal volume is 0.6 mL. The ports were scanned by a Siemens CT scanner (SOMATOM Definition Edge, Siemens Medical Solutions USA, Malvern, PA) with a resolution of 0.98 mm × 0.98 mm and a slice thickness of 3 mm.

### 2.2 IDD acquisition

IDDs of 100‐, 160‐, and 200‐MeV pencil beamlets with and without a chemo‐port were first acquired. Measurements were taken with a MLIC (Giraffe; IBA Dosimetry, Schwarzenbruck, Germany), from a clinical cyclotron system (ProBEAM, Varian Medical System, Palo Alto, CA). The 1σ spot size of the beam at iso in the air was 5.0, 3.9, and 3.5 mm for the 100‐, 160‐, and 200‐MeV pencil beamlets, respectively.

IDDs were obtained with the chemo ports in the beamline, at both vertical and lateral orientations. Due to the round shape of the ports, their setup for the lateral IDDs was challenging. We used a metal rod and, inserted one end into the stem of the port, and taped the other end to solid water (Figure 1c). The stem was on the side of the port and connected to the central cavity. The stick was just properly fitted to the stem structure without going into the main part of the port. The port structures were symmetric about the stem axis, so lateral and vertical orientations could be achieved by simply rotating the port about the stem. For the measurement, the port was aligned with the in‐room laser system. We used orthogonal pencil beamlets to determine the WETs and SPRs of the main structures, and then applied the results to the corresponding structures of the chemo‐port in the patient CT scan. The SPRs of the structures are independent of beam angles.

A 3‐cm‐thick lead plate with a 5‐mm diameter bore was used as a collimator to reduce the spot size. The central axis of the bore was aligned with the in‐room laser to allow the beamlet to pass through. The tolerance for the alignment of lasers and beam central axis was 2 mm and 1°. With the collimator in place, only IDDs with 100‐ and 160‐MeV beamlets were measured, as the range of 200‐MeV beamlets is more than the thickness of the collimator.

### 2.3 WET of chemo‐port

After the IDD was acquired, the ES was extracted by decomposing the IDD into mono‐energetic IDDs as described by Yao et al.^4^ Without a chemo‐port, the open beam IDD had a single‐peak Gaussian ES.^4^ With a chemo‐port, the proton energy decreased, and the spectrum showed multiple peaks as the beamlet passed through the complex internal structure of the port. By analyzing the shifts of the corresponding peaks, the WETs of the chemo port structures can be calculated:

(1) $$\mathrm{WE}T_{\mathrm{structure}}=R_{90}\left(E_{\mathrm{open}}\right)-R_{90}\left(E_{\mathrm{structure}}\right),$$

where E_open_ is the energy at the peak in the extracted ES from the IDD without chemo‐port, and E_structure_ is the energy at the corresponding peak to the chemo‐port structure in the extracted ES from the IDD with the chemo‐port. R_90_(E) is the distal range of mono‐energetic (E) protons at 90% of the Bragg peak in water.

### 2.4 SPR of chemo‐port

The SPR was determined by the WET and the geometric thickness (GT) of the structure excluding the air cavity.

(2) $$\mathrm{SP}R_{\mathrm{structure}}=\mathrm{WE}T_{\mathrm{structure}}/GT_{\mathrm{structure}}$$

The GT was measured from the CT scan of the port under a specific window and level of the CT image. As the measured GT depends on the window and level, this causes uncertainty in the GT measurement and consequently SPR determination. However, the WET is independent of the window and level settings, and dose deposition is proportional to the stopping power multiplied by the GT, that is, the WET multiplied by the stopping power of water.

## 3 RESULTS

**FIGURE 2:**
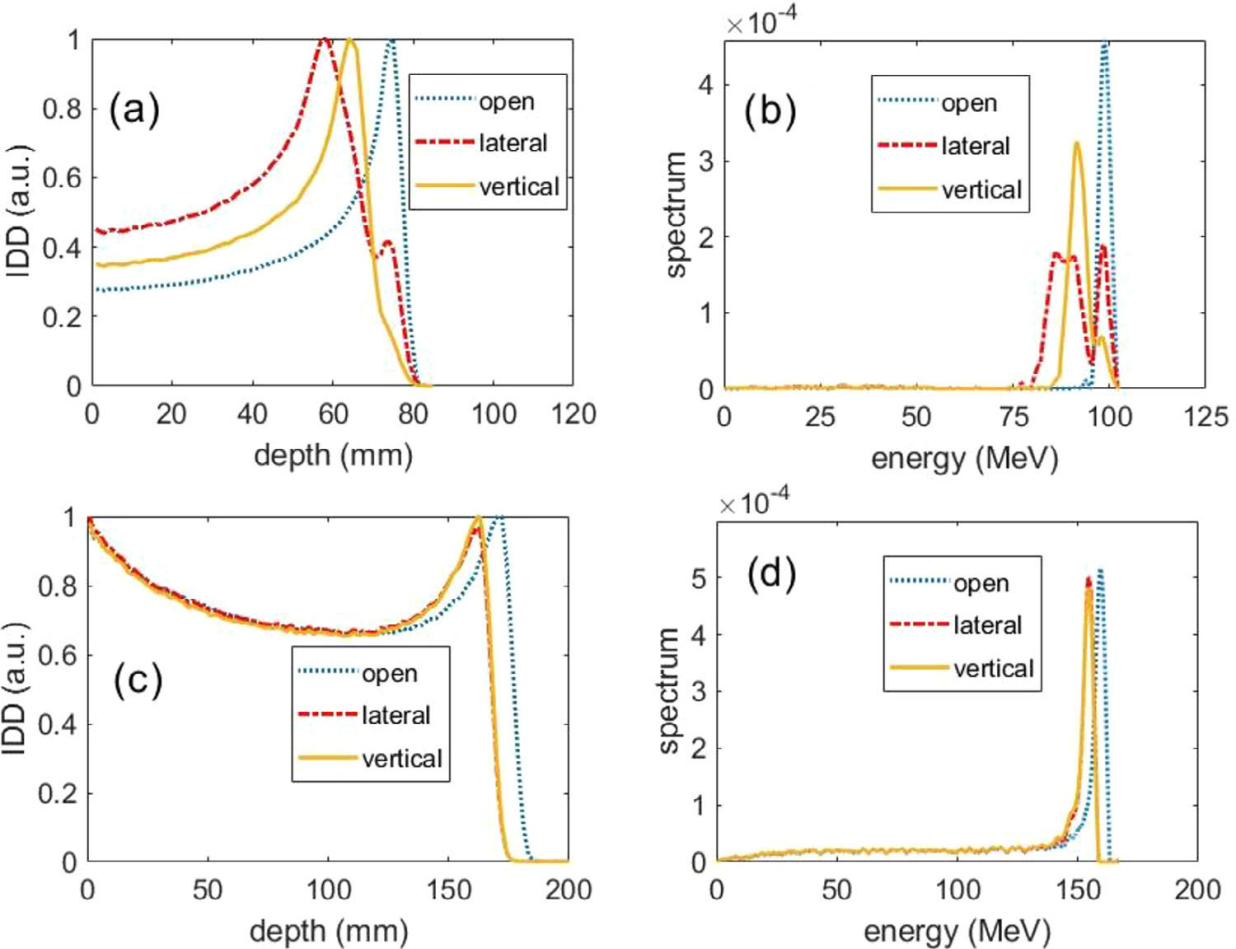
Measured integrated depth dose (IDD) (left) and extracted energy spectra (right) from 100 MeV pencil beamlet without collimator (top) and from 160 MeV pencil beamlet with collimator (bottom) for the slim chemo‐port.

Figure 2 shows the IDD and the extracted energy spectra of 100 and 160 MeV with and without a collimator for the slim chemo port. For the 100‐MeV beam in the lateral orientation without a collimator, there were 3 peaks in the extracted ES: the high energy peak matched the open beam peak, so that was due to protons missing the port entirely; the middle peak was from the protons traversing through the air cavity and the port walls; the low energy peak corresponds to the protons traversing through the top structure of the port, which contains the most structural material traversed by the proton beam. The information was clearly displayed in the extracted ES, but not in the IDD. For the vertical beam, the extracted ES displayed 2 peaks: the lower peak had the same energy as the open beam, and the higher peak corresponded to the majority of protons passing through the port.

For the 160 MeV beam with a 5‐mm diameter collimator, the IDDs had big tails, and the ES had continuous low‐energy components. This is due to energy loss when the protons interact with the wall of the collimator bore. The extracted ES had only one peak corresponding to the port's structure along the air cavity laterally for the lateral beam and vertically for the vertical beam. This is expected, as the collimator bore size was selected to be much smaller than the port.

**FIGURE 3:**
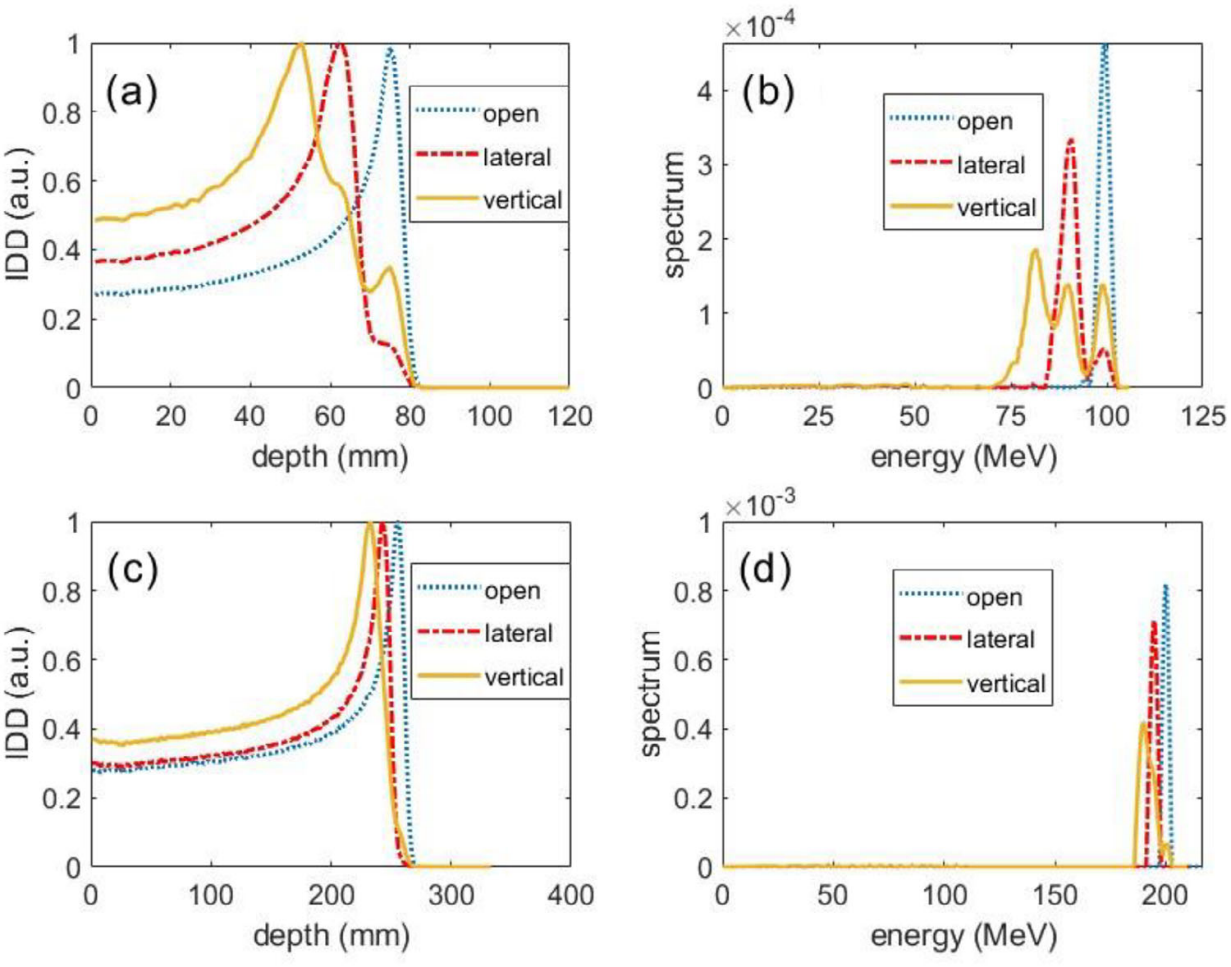
Measured integrated depth dose (IDD) (left) and the extracted energy spectra (right) from 100 MeV pencil beamlet (top) and from 200 MeV pencil beamlet (bottom) without collimator for the isp chemo‐port.

Figure 3 shows the IDD and the extracted energy spectra of 100‐ and 200 MeV beamlets without a collimator. For the 100 MeV lateral beam, there were 2 peaks in the extracted ES: the lower peak had the same energy as the open beam, and the higher peak corresponded to the majority of protons passing through the port. For the vertical beam, the extracted ES displayed 3 peaks: the right peak was from those protons through the air because the peak corresponded to the same energy of the open beam; the middle peak was from the protons traversing through the top structure and air cavity; the left peak was from the protons traversing through the wall structure of the port because (1) the middle peak was fewest in counts and the estimated proton fluence (in Gaussian distribution) passing through the central structure (about 4.5 mm in radius) of the port is less than that passing through the remaining structures (wall thickness about 3.5 mm), and (2) the energy loss was less by going through the air cavity structure than the wall structure of the port.

The determined WETs of the port structures by the ES are listed in Table 1. Ideally, the determined WETs are energy‐independent. In Table 1a, the max difference of the WET was 1.1 mm, which happened in the lateral structure along the air cavity. In Table 1b, the max difference was 0.5 mm. As a comparison, Table 2 lists the WETs determined as the shift of the primary peak in the beam IDD with the chemo‐port from the open beam IDD. The max difference was 8 mm happened in the lateral beam along the air cavity of the small port, and 2 mm otherwise.

**TABLE 1:** Determined WET from the shifts in ES peaks for (a) slim chemo‐port and (b) isp chemo‐port.

(a)						
	100 MeV beam w/o collimator			160 MeV w collimator		
Structure	Open E (MeV)	E w/port (MeV)	WET (mm)	Open E (MeV)	E w/port (MeV)	WET (mm)
Lateral along top	98.8	86.5	16.0	NA	NA	NA
Lateral along air cavity	98.8	90.8	10.6	160	154.6	9.5
Vertical along air cavity	98.8	91.9	9.4	160	154.7	9.3
Vertical along wall	98.8	93.3	7.4	NA	NA	NA

(b)						
	100 MeV beam w/o collimator			200 MeV w/o collimator		
Structure	Open E (MeV)	E w/port (MeV)	WET (mm)	Open E (MeV)	E w/port (MeV)	WET (mm)
Lateral along air cavity	98.8	90.5	10.9	200.1	194.8	11.4
Vertical along air cavity	98.8	89.9	11.7	200.1	194.5	11.8
Vertical along wall	98.8	82.1	21.8	200.1	190.0	21.7

Abbreviation: WET, water equivalent thickness.

**TABLE 2:** Determined WET from the shifts in IDD peaks for (a) slim chemo‐port and (b) isp chemo‐port.

(a)						
	100 MeV beam w/o collimator			160 MeV w collimator		
Structure	Open	w/port	WET	Open	w/port	WET
Lateral along air cavity (mm)	75	58	17	171	162	9
Vertical along air cavity (mm)	75	64	11	171	162	9

(b)						
	100 MeV beam w/o collimator			200 MeV w/o collimator		
Structure	Open	w/port	WET	Open	w/port	WET
Lateral along air cavity (mm)	75	62	13	256	243	13
Vertical along air cavity (mm)	75	53	22	256	233	23

Abbreviation: WET, water equivalent thickness.

Finally, with window = 500 HU and level = 1000 HU in the CT, we obtained the GT of the small port 10, 7, and 7 mm along the lateral top, lateral air cavity, and vertical air cavity, respectively. From Table 1a, we obtained SPR = 1.6, 1.5, and 1.3 of the materials along the lateral top, lateral air cavity, and vertical air cavity, respectively. For the isp port, with the same window and level, the GT was 7.6 and 7.7 mm along the lateral and vertical directions passing through the center of the air cavity. From Table 1b, we had SPR = 1.4, 1.5 along the lateral and vertical. The SPR = 21.8/9.8 mm = 2.2 along the wall from the vertical beam because there was a high‐density ring on the top of the wall. Here, 9.8 mm was the height of the isp port.

## 4 DISCUSSION

We introduced our novel approach to determine the WET of chemo‐ports by extracting the ES from the measured IDDs and analyzing the shift of the ES peaks. Often, chemo‐ports in breast patients are similar in size to a proton pencil beamlet, with complicated structures. We showed that complicated structures could be distinguished and extracted from measured IDDs. From the CT image of the port, we measured the geometry of the port and then estimated the SPR of the material of the port. The estimated SPR was about 1.5. Comparatively, Athar measured the SPR of the common materials used in the PowerPort chemo‐ports,^8^ where the material was in the shape of a slab and the SPR was determined from the IDD shift with and without the slab. The SPR was 1.26 for the main material in the slim port, and 1.35 for the isp port.^8^ Our estimated SPR depends on the window/level of the CT image for the GT, and thus, the estimated SPR can deviate from the truth but will not affect the dose calculation. This is because the SPR and the GT are associated, but the dose is proportional to their product, that is, WET, which is independent of the window/level. Of course, a proper window/level is appreciated, especially when the port is in the patient's body. In this case, the port is surrounded by tissues, not air.

In the conventional approach, the WET is determined from the shift in the peaks of the IDDs with and without the port. A collimator is usually needed to reduce the spot size of the pencil beam so that the beam passes the expected structure, and the resultant IDD has a sharp peak. From our work, we found the IDD was sensitive to the setup of the collimator and it was challenging to align the collimator to the pencil beam. Even if the alignment is well done, a majority of protons will interact with the collimator wall, resulting in a different shape of Bragg peak. In Table 1a, the collimator introduced up to 1.1 mm difference in the WET between energy 100 and 160 MeV, while in Table 1b without the collimator, the difference in WET was up to 0.5 mm between energy 100 and 200 MeV. In contrast, without the collimator, the shape of IDD is less sensitive to the alignment of the chemo‐port and laser as the spot size is comparable to the size of the chemo‐port. Similar phenomena were observed from the open beam (without the chemo‐port) IDDs: for 100 MeV, the Bragg peak was shifted −0.1 MeV from 98.8 MeV without collimator to 98.7 MeV with the collimator, and for 160 and 200 MeV, the shifts were −0.45 and −4.0 MeV.

As a result of the conventional approach, the max difference in WET was 8 mm (Table 2a). This is because the highest peak in the IDD of the 100 MeV lateral beam did not correspond to the port structure along the air cavity but along the top structure. It is not easy to distinguish the corresponding structure from the IDD, but it can be determined from the extracted ES. Furthermore, the max difference of the other WET in Table 2 was 2 mm, much larger than 0.5 mm from our approach.

Our MLIC has a 1.86‐mm physical resolution. However, the pencil beams used in our study (100, 160, and 200 MeV) have more than 6‐mm distal regions. As long as the distal region is adequately sampled, the ES extracted by our algorithm remains reliable. We also decomposed the measured IDDs using 1‐ and 0.1‐mm resolutions and obtained consistent energy spectra in each case.

We scanned the chemo‐port when the port was positioned upright on the CT couch, allowing us to measure both the lateral and vertical geometric thicknesses in the central slice, as displayed in Figure 1a,b in the text. The scanning resolution was 0.98 mm × 0.98 mm, and the slice thickness was 3.0 mm. The slice thickness has a minimal effect on the geometric thickness estimation because the diameters of the ports were > 20 mm, making it straightforward to determine the central slice. Furthermore, we can scan the chemo‐port sample with even high‐resolution CT and use the physically measured size of the shape to guide the determination of the appropriate window/level for CT image display.

Regarding the setup and range uncertainties used for robust optimization, we do not treat the chemo‐port as special, but rather as a part of the body. Also, in our practice, we make efforts to avoid the chemo‐port being in the beam path.

Charyyev et al measured the SPRs of small metal beads (Al, Ti, and CoCr) with a pixelized spectrum detector (also called a microdosimeter) AdvaPIX‐TPX3.^9^ The ES of protons after traversing the object was obtained. They showed a high accuracy of the measurement validated by the vendor, which provided nominal densities. However, the microdosimeter requires a proton dose rate as low as 10^4^ protons/cm^2^/s, about 0.3% of the lowest dose rate deliverable in clinic mode, limiting the clinical use of the detector. Our method does not have this limitation. Furthermore, the large size of MLIC allows us to collect large‐angle scattered protons to catch the structure information of the scatterer by delivering one beamlet.

## 5 CONCLUSION

The WET of a chemo‐port can be accurately determined from the energy spectra of a proton beamlet traversing through the chemo‐port. The internal structures of the chemo‐port can be distinguished from the peaks in the energy spectra, and thus, a collimator is not necessary for a specific structure. Our approach can practically be used with clinical equipment.

## AUTHOR CONTRIBUTIONS

W.Y. designed the measurement, extracted the energy spectrum, and conducted data analysis. R.G. measured IDDs and drafted the manuscript. S.O. measured IDDs and edited the manuscript. S.M. improved the manuscript.

## CONFLICT OF INTEREST STATEMENT

The authors declare no conflicts of interest.

## Data Availability

The data that support the findings of this study are available from the corresponding author upon reasonable request.

## References

[1] Klaassen L , Petoukhova AL , Habraken SJM , et al. Effect of breathing motion on robustness of proton therapy plans for left‐sided breast cancer patients with indication for locoregional irradiation. Acta Oncol. 2021;60(2):222‐228. 33269958 10.1080/0284186X.2020.1825800

[2] Taylor CW , Zhe W , Macaulay E , Jagsi R , Duane F , Darby SC . Exposure of the heart in breast cancer radiation therapy: a systematic review of heart doses published during 2003 to 2013. Int J Radiat Oncol Biol Phys. 2015;93:845‐853. 26530753 10.1016/j.ijrobp.2015.07.2292

[3] Aznar MC , Duane FK , Darby SC , Wang Z , Taylor CW . Exposure of the lungs in breast cancer radiotherapy: a systematic review of lung doses published 2010–2015. Radiother Oncol. 2018;126:148‐154. 29246585 10.1016/j.radonc.2017.11.022 PMC5807032

[4] Yao W , Farr JB . Technical note: extraction of proton pencil beam energy spectrum from measured integral depth dose in a cyclotron proton beam system. Med Phys. 2021;48:7504‐7511. 34609749 10.1002/mp.15261

[5] BD . “PowerPort ClearVUE Implantable Port product brochure.” *PowerPort ClearVUE Implantable Port product brochure* . 2018. March 2023. https://www.bd.com/content/dam/bd‐assets/naperipheral‐intervention/web‐assets/us/documents/BD‐88828v3_PPCV_BRO_6_22_23_23‐FINAL.pdf

[6] PowerPort™ ClearVUE™ isp Implantable Port. n.d. 2023. https://www.bd.com/en‐us/products‐and‐solutions/products/product‐page.1608062#overview

[7] PowerPort™ ClearVUE™ Slim Implantable Port. n.d. 2023. https://www.bd.com/en‐us/products‐and‐solutions/products/product‐families/powerport‐clearvue‐slim‐implantable‐port#overview

[8] Athar Basit . Proton beam evaluation of common implantable port materials. IO Leaning. 2021;9:E1‐E4. https://www.hmpgloballearningnetwork.com/site/iolearning/white‐papers/proton‐beam‐evaluation‐common‐implantable‐port‐materials#:~:text=Sometimes%2C%20previously%20implanted%20medical%20devices%2C%20such%20as%20ports%2C,a%20proton%20beam%20and%20measure%20

[9] Charyyev S , Chang C‐W , Harms J , et al. A novel proton counting detector and method for the validation of tissue and implant material maps for Monte Carlo dose calculation. Phys Med Biol. 2021;66:1361‐6560. 10.1088/1361-6560/abd22e 33296888

